# Large–Scale Transposon Mutagenesis Reveals Type III Secretion Effector HopR1 Is a Major Virulence Factor in *Pseudomonas syringae* pv. *actinidiae*

**DOI:** 10.3390/plants12010141

**Published:** 2022-12-27

**Authors:** Takako Ishiga, Nanami Sakata, Giyu Usuki, Viet Tru Nguyen, Kenji Gomi, Yasuhiro Ishiga

**Affiliations:** 1 Faculty of Life and Environmental Sciences, University of Tsukuba, 1-1-1 Tennodai, Tsukuba 305-8572, Ibaraki, Japan; 2 Western Highlands Agriculture and Forestry Science Institute, 53 Nguyen Luong Bang Street, Buon Ma Thuot City 630000, Vietnam; 3 Faculty of Agriculture, Kagawa University, Miki 761-0795, Kagawa, Japan

**Keywords:** *Pseudomonas syringae* pv. *actinidiae*, bacterial canker of kiwifruit canker, type three secretion system, stomatal-based defense, HopR1

## Abstract

Bacterial canker of kiwifruit caused by *Pseudomonas syringae* pv. *actinidiae* (Psa) is a serious threat to kiwifruit production worldwide. Four biovars (Psa biovar 1; Psa1, Psa biovar 3; Psa3, Psa biovar 5; Psa5, and Psa biovar 6; Psa6) were reported in Japan, and virulent Psa3 strains spread rapidly to kiwifruit production areas worldwide. Therefore, there is an urgent need to develop critical management strategies for bacterial canker based on dissecting the dynamic interactions between Psa and kiwifruit. To investigate the molecular mechanism of Psa3 infection, we developed a rapid and reliable high-throughput flood-inoculation method using kiwifruit seedlings. Using this inoculation method, we screened 3000 Psa3 transposon insertion mutants and identified 91 reduced virulence mutants and characterized the transposon insertion sites in these mutants. We identified seven type III secretion system mutants, and four type III secretion effectors mutants including *hopR1*. Mature kiwifruit leaves spray-inoculated with the *hopR1* mutant showed significantly reduced virulence compared to Psa3 wild-type, indicating that HopR1 has a critical role in Psa3 virulence. Deletion mutants of *hopR1* in Psa1, Psa3, Psa5, and Psa6 revealed that the type III secretion effector HopR1 is a major virulence factor in these biovars. Moreover, *hopR1* mutants of Psa3 failed to reopen stomata on kiwifruit leaves, suggesting that HopR1 facilitates Psa entry through stomata into plants. Furthermore, defense related genes were highly expressed in kiwifruit plants inoculated with *hopR1* mutant compared to Psa wild-type, indicating that HopR1 suppresses defense-related genes of kiwifruit. These results suggest that HopR1 universally contributes to virulence in all Psa biovars by overcoming not only stomatal-based defense, but also apoplastic defense.

## 1. Introduction

*Pseudomonas syringae* induces a variety of symptoms such as leaf spots, cankers, galls, wilt, and blights on different plants, and can be classified into more than 60 pathovars (pv.) based on their host plant specificities and disease symptoms [1,2]. *P. syringae* pv. *actinidiae* (Psa), a causal agent of bacterial canker of kiwifruit, is an economically important pathogen worldwide. Psa was first found in Japan and causes severe damage to kiwifruit plants and decreases yield [3,4]. Psa strains are organized into five biovars including biovar 1, 2, 3, 5, and 6 based on biochemical, pathogenicity, and molecular characteristics [5,6]. Psa3 strains with enhanced virulence were reported in 2008 in Italy, and a pandemic spread rapidly to kiwifruit production areas worldwide [3]. Major control strategies include chemical treatments such as copper fungicides and antibiotics [7], but they are associated with potential risks such as induction of Psa resistance strains, phytotoxicity, and fruit chemical residues [8,9,10]. Resistance breeding is another control strategy, but cultivar development is slow [11]. Therefore, it is necessary to investigate Psa infection mechanisms to develop effective and sustainable control strategies.

*P. syringae* has two lifestyles, an epiphytic phase on the plant leaf surface and an endophytic phase in the apoplastic space [2,12]. The bacterium generally enters host tissues through natural openings such as stomata and multiplies in the apoplast to cause disease in nature [13]. Plants have developed monitoring systems that recognize potential invading pathogens and activate a wide range of immune responses to defend themselves [14,15]. The first line of plant defense against invading bacterial pathogens is pathogen-associated molecular pattern (PAMP)-triggered immunity (PTI), which recognizes conserved molecules such as flagellin and elongation factor Tu (EF-Tu) of the invading bacterial pathogens using plant pattern-recognition receptors (PRRs), such as FLS2 and EFR, respectively [16,17,18]. After recognition of invading bacterial pathogens with PRRs, plants activate many defense mechanisms including stomatal-based defense, rapid oxidative burst, restriction of nutrient transfer from the cytosol to the apoplastic space, the accumulation of antimicrobial compounds including phytoalexins, and the activation of hormone-mediated signaling pathways leading to defense responses [12,19,20,21,22,23,24,25,26,27]. Stomatal-based defense to restrict bacterial pathogen entry by stomatal closure is one of the earliest responses in PTI [12,28,29,30]. However, successful bacterial pathogens have evolved to acquire multiple virulence factors such as phytotoxins and type III secretion system (T3SS) effectors (T3SEs) to overcome PTI and stomatal-based defense [31,32]. The T3SS translocates bacterial effector proteins into host cells, which is central for pathogenesis in diverse plant-bacterial interactions [33]. *P. syringae* has canonical tripartite pathogenicity islands (T-PAIs), with the *hrp* and *hrc* gene clusters encoding the T3SS flanked by both the conserved effector locus (CEL) such as *hopM1* and *avrE*, and the exchangeable effector loci (EEL) [34]. T3SS effectors (T3SEs) have functional overlap by targeting different steps in PTI [35]. Plant immunity suppression and creation of an aqueous apoplast are two main features of *P. syringae* leaf infection [36,37]. These virulence strategies, supported by the right environmental conditions (for example, high humidity) are a prerequisite for successful endophytic growth [37].

To identify virulence related genes in bacterial pathogens, forward screens have been conducted in previous studies [38,39,40]. Generation and characterization of transposon insertion mutation libraries is one of the most efficient screens. In previous studies, Tn*5* mutant bacterial pathogen forward screenings were performed with various plants and inoculation methods to identify reduced virulence mutants. Plants have been inoculated with *P. syringae* using syringe pressure infiltration, vacuum infiltration, sprays, and dips to investigate the molecular basis of plant-pathogen interactions [41]. Syringe pressure infiltration is the most commonly used method [11]. Conventional soil-grown plant inoculation assays including stem puncture inoculation, syringe pressure infiltration, and spray inoculation were used to study Psa and kiwifruit interactions [11,42,43]. However, to functionally investigate *P. syringae*-plant molecular interactions, the inoculation assay should mimic natural infection and be suitable for high-throughput assays. We developed a rapid and reliable flood-inoculation method using kiwifruit seedlings that had been grown in Murashige and Skoog (MS) medium [44] based on flood-inoculation using *P. syringae* pv. *tomato* DC3000 [45,46,47].

Here, we developed a high-throughput screening method using kiwifruit seedlings grown in MS medium to investigate Psa virulence mechanisms. Using this screening method, we screened 3000 Psa3 transposon insertion mutants and identified 91 virulence related genes. We focused on the T3SS effector HopR1 identified from this screening, and revealed that HopR1 is a major Psa virulence factor. Here, we demonstrated that HopR1 contributes to Psa virulence on kiwifruit plants in both early and later stages of the infection process by suppressing stomatal-based and apoplastic defenses.

## 2. Results

### 2.1. Isolation of Psa3 Mutants with Reduced Virulence

**Figure 1:**
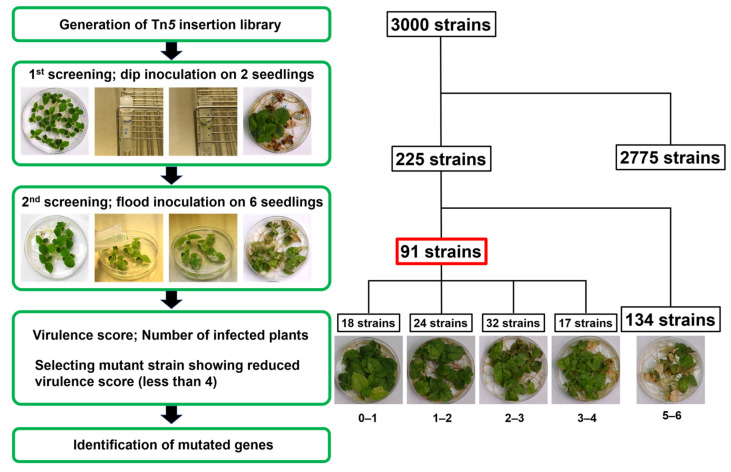
The workflow to identify *Pseudomonas syringae* pv. *actinidiae* biovar 3 virulence factor mutants. The transposon was introduced into Psa3-15; Psa biovar 3 (MAFF 212115) by conjugation with *E. coli* S17-1, which possessed pBSLC1, and the insertion region was integrated into the Psa3-15 chromosome randomly. To assay for disease on kiwifruit seedlings (first screening), 3000 individual Psa3-15 Tn*5* mutants were dip-inoculated onto two kiwifruit plants, which were about 3 to 4 weeks old. Then, disease symptoms were observed up to 14 days post-inoculation (dpi). Mutant strains that caused little or no leaf spot were chosen for further analyses. A total of 225 mutants showed reduced virulence on kiwifruit and were flood-inoculated on six kiwifruit seedlings. A virulence score was assigned by counting infected seedlings, and each experiment was repeated at least three times. Mutant strains with a virulence score less than 4 were chosen for further analyses. A total of 91 mutants showed reduced virulence on kiwifruit and the mutated genes were determined. To identify the mutated genes, the resultant DNA was ligated with T4 DNA ligase, then introduced into *E. coli* DH5α competent cells. A *Pseudomonas* Genome DB BLAST search (http://www.pseudomonas.com/blast/setnblast, accessed on 14 February 2020) was utilized to identify the function of the mutated genes.

We conducted a screening for mutants with reduced virulence of Psa biovar 3 (MAFF 212115) (Supplementary Figure S1). At this point, two kiwifruit seedlings were dip-inoculated with 3000 individual Psa3 Tn*5* mutants for initial screening, and we observed disease symptoms up to 14 days post inoculation (dpi). Disease symptoms on kiwifruit seedlings inoculated with Psa3 wild-type (WT) included leaf spot and necrosis (Figure 1). A total of 225 mutants showed reduced virulence in the initial screening, and flood-inoculations were conducted with six kiwifruit seedlings for a secondary screening. Six seedlings were evaluated for virulence score by counting infected seedlings, and we observed disease symptoms at 7 dpi. This secondary seedling screening was repeated at least three times. Mutant strains showing a virulence score of less than 4 were chosen for further analyses. A total of 91 mutants showed reduced virulence on kiwifruit (Figure 1, Supplementary Figure S2–S4). These Tn*5* mutants were also tested for their ability to grow on minimal medium containing mannitol and fructose (MMMF). Nine mutants were partial auxotroph because they did not grow in MMMF (Table 1).

### 2.2. Identification of Genes Disrupted by Tn5 Insertions

Next, we identified the Tn*5* insertion sites of 91 mutant strains that showed reduced virulence. Our results suggest that various virulence factors are needed for the infection process by Psa3 (Table 1). Well-known virulence factors included those involved in T3SS and T3SEs and cell motility/chemotaxis/adhesion, along with transporters. Several genes were identified as a hypothetical protein, whose function was unknown. Furthermore, we identified regulators, including signal transduction regulators involved in two component systems, and transcription factors, which are required for survival during environmental stress conditions. Other Tn*5* insertions were located in genes related to primary metabolism such as nucleotide metabolism, amino acid metabolism, carbohydrate metabolism, and lipid metabolism (Table 1).

Three partial auxotroph mutants in amino acid metabolism had Tn*5* insertions in genes encoding serine hydroxymethyltransferase (GlyA-2), ketol-acid reductoisomerase, and homoserine O-acetyltransferase (MetX). Moreover, three partial auxotroph mutants in carbohydrate metabolism had Tn*5* insertions in genes encoding 6-phosphogluconolactonase (Pgl), aminoglycoside phosphotransferase, and aconitate hydratase. Further, two partial auxotroph transporter mutants and one in type IV secretion system had Tn*5* insertions in genes encoding sugar ABC transporter ATP-binding protein, autotransporting lipase, GDSL family, and protein disulfide-isomerase (Table 1).

The mutants that showed complete pathogenicity impairment on kiwifruit seedlings (virulence score 0) had Tn*5* insertions in genes encoding serine hydroxymethyltransferase, ketol-acid reductoisomerase, T3SS protein HrcU, T3SS protein HrcJ, T3SS protein HrcR, and T3SS protein HrpR. The category of ‘‘type III secretion system” and ‘‘amino acid metabolism and transport’’ were enriched in these genes (Table 1).

### 2.3. Bacterial Growth of the Virulence Mutants in Plant Tissue

To determine whether the reduction in disease symptom production was correlated with reduced multiplication in plant tissue, 91 mutants were analyzed for their ability to grow in kiwifruit seedlings after flood-inoculation. The Psa3-15 WT multiplied from 6 to 6.5 (Log [CFU/mg]), and all the mutants except six (TZ32, TAc09, TBh17, TBp22, TBu09, and TBw17) exhibited a significant growth reduction relative to Psa3-15 WT on kiwifruit seedlings (Supplementary Figure S5A–C).

### 2.4. Hypersensitive Response Cell Death Assay with T3SS/T3E Mutants

The T3SS/T3SEs had the greatest number of mutant strains showing reduced virulence (Table 2 and Table S2). We identified five mutant strains (TD35, TJ40, TAh32, TAl39, and TBi11) including duplicates (as *hrpR* mutants, TD35 and TAl39) of the T3SS, two mutant T3SS helper strains (TAw18 and TAd35), and four T3SE mutant strains (TM33, TR23, TBr13, and TBw35) (Supplementary Table S2). The T3SS mutants showed complete pathogenicity impairment on kiwifruit seedlings (virulence score 0). T3SS locaters TAw18 and TAd35 had Tn*5* insertions in genes encoding type III helper protein HrpK1 and lytic transglycosylase (HrpH). Previous studies showed that both of these genes encoding translocators of T3SS effectors elicited reduced hypersensitive response (HR) cell death [50,51]. To confirm this, T3SS/T3SE mutants were assayed for their ability to elicit macroscopic tissue collapse indicative of HR on nonhost tobacco leaves. Inconsistent with virulence scores, the T3SS mutants failed to cause HR cell death, except for TAw18 and TAd35. TAw18 elicited reduced HR cell death compared to Psa3 WT, while TAd35 showed the same levels of HR cell death as Psa3 WT.

All T3SE mutants induced HR cell death (Supplementary Figure S6) and did not show complete pathogenicity impairment on kiwifruit seedlings (Supplementary Table S2). TM33, TR23, TBr13, and TBw35 had Tn*5* insertions in genes encoding type III effectors HopZ3, HopR1, HopAC1, and AvrE1, respectively. Despite the HR phenotype, TR23 exhibited a significantly reduced virulence score of 0.67. This mutant strain had Tn*5* insertions in genes encoding the type III effector HopR1.

### 2.5. HopR1 Contributes to Psa3 Virulence

**Figure 2:**
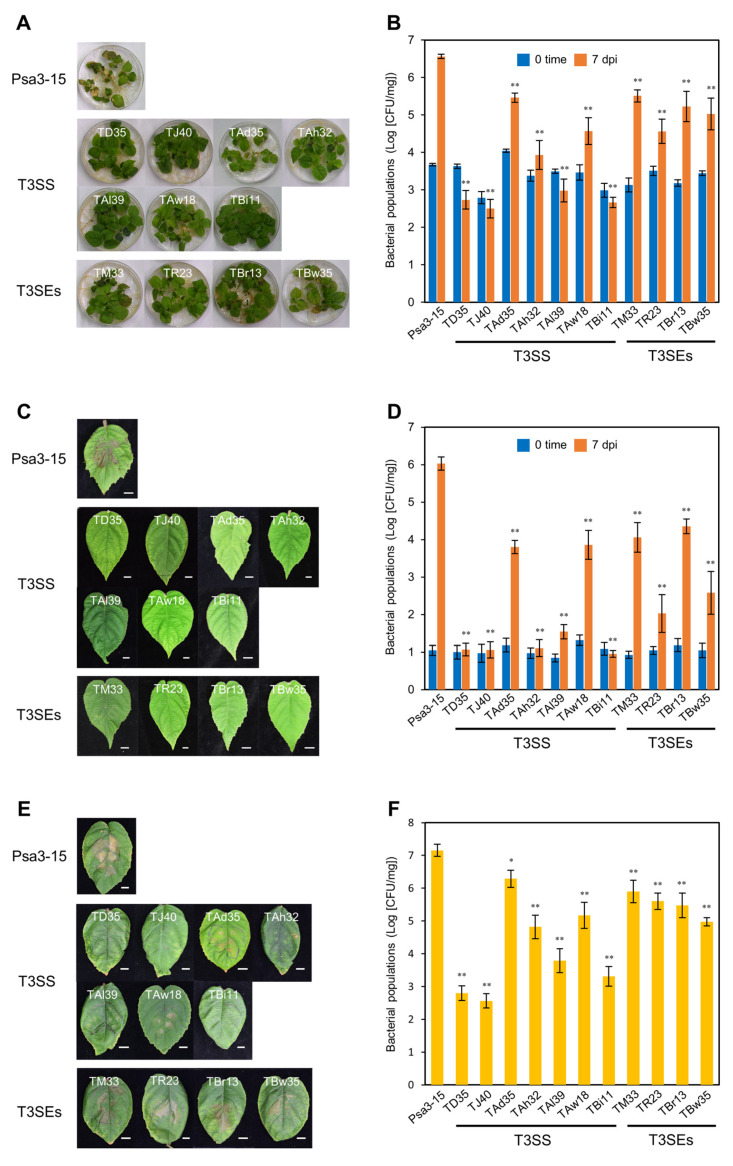
Disease symptoms and bacterial populations in kiwifruit inoculated with *Pseudomonas syringae* pv. *actinidiae* biovar 3 WT and type III secretion mutants. Disease symptoms (**A**) and bacterial populations (**B**) in seedlings flood-inoculated with *P. syringae* pv. *actinidiae* biovar 3 (Psa3) WT and type III secretion mutants (1 × 10^8^ CFU/mL) containing 0.025% SilwetL-77. Disease symptoms (**C**) and bacterial populations (**D**) in kiwifruit spray-inoculated with Psa3 WT and type III secretion mutants (1 × 10^8^ CFU/mL) containing 0.025% SilwetL-77. Disease symptoms (**E**) and bacterial populations (**F**) in kiwifruit syringe-inoculated with Psa3 WT and the type III secretion mutants (5 × 10^6^ CFU/mL). The leaves were photographed at 7 dpi. Vertical bars indicate the standard error for three independent experiments. Asterisks indicate a significant difference between Psa3 WT and the type III secretion mutants in a *t* test (* *p* < 0.05, ** *p* < 0.01). Scale bar shows 1 cm.g.

Growth of the T3SS/T3SE mutants did not differ significantly at 24 h based on a growth curve assay (Supplementary Figure S7). To investigate whether the T3SS/T3SEs play an important role in causing disease on kiwifruit seedlings, we flood-inoculated kiwifruit seedlings with Psa3 WT and the T3SS/T3SE mutants. All kiwifruit seedlings inoculated with the T3SS/T3SE mutants exhibited less leaf spot and necrosis symptoms at 7 dpi (Figure 2A). Kiwifruit seedlings inoculated with T3SS mutants showed no symptoms, whereas kiwifruit seedlings inoculated with T3SS locaters and T3SEs showed fewer symptoms compared to Psa3 WT. Seedlings inoculated with the TR23 (*hopR1* mutant) showed the least number of symptoms among the T3E mutants. We also investigated whether the T3SS/T3SEs contribute to bacterial multiplication in kiwifruit seedlings. T3SS/T3SE mutant populations were significantly reduced compared to Psa3 WT at 7 dpi (Figure 2B). T3SS mutant populations were approximately 10,000-fold less compared to Psa3 WT at 7 dpi (Figure 2B). T3SS locaters and T3E mutant populations were mildly reduced compared to the T3SS mutant strains. TR23 (*hopR1* mutant) populations showed the greatest reduction among the T3E mutants. These results indicate that HopR1 contributes to Psa3 virulence on kiwifruit seedlings.

To investigate if HopR1 contributes to Psa3 virulence on adult kiwifruit plants, we next spray-inoculated soil-grown kiwifruit plants with Psa3 WT and the T3SS/T3SE mutants. Mature kiwifruit leaves inoculated with Psa3 WT showed severe symptoms such as leaf spots and necrosis (Figure 2C). However, severe symptoms were not observed on T3SS/T3SE-inoculated leaves. Consistent with disease development, the T3SS/T3SE mutant populations were significantly reduced compared to Psa3 WT (Figure 2C). The T3SS mutant populations were approximately 100,000-fold less compared to Psa3 WT at 7 dpi (Figure 2D). Consistent with flood-inoculation, the populations of T3SS locaters and T3Es were mildly reduced compared to the T3SS mutants. Interestingly, TR23 inoculated leaves showed a significant reduction in disease development among the T3E mutants (Figure 2C), and TR23 populations were approximately 10,000-fold less compared to Psa3 WT (Figure 2D). These results indicate that HopR1 also contributes to Psa3 virulence on mature kiwifruit plants. 

In nature, *P. syringae* has two lifestyles, an epiphytic phase on the plant leaf surface and an endophytic phase in the apoplastic space where the bacterium multiplies to cause disease [13,14]. To determine whether HopR1 is important for apoplastic multiplication, we next conducted syringe-infiltration. Kiwifruit leaves inoculated with Psa3 WT showed necrosis at 7 dpi. Kiwifruit leaves inoculated with the T3SS mutants showed no necrosis compared to Psa3 WT (Figure 2E). However, kiwifruit leaves inoculated with the T3SS locaters and T3SEs showed less necrosis compared to Psa3 WT (Figure 2E). We next examined bacterial multiplication in kiwifruit inoculated leaves. The T3SS mutant populations were significantly smaller compared to Psa3 WT at 7 dpi (Figure 2F). The populations of T3SS locaters and T3Es were mildly reduced compared to the T3SS mutants (Figure 2F). Interestingly, there were no significant differences in disease development and bacterial populations between TR23 and the other T3E mutants (Figure 2E,F). These results indicate that HopR1 contributes to Psa3 virulence, but the contribution differs depending on the inoculation method. These results indicate that HopR1 might contribute to Psa3 virulence at the plant surface or before apoplastic multiplication to cause disease.

### 2.6. HopR1 Universally Contributes to Virulence in All Psa Biovars

**Figure 3:**
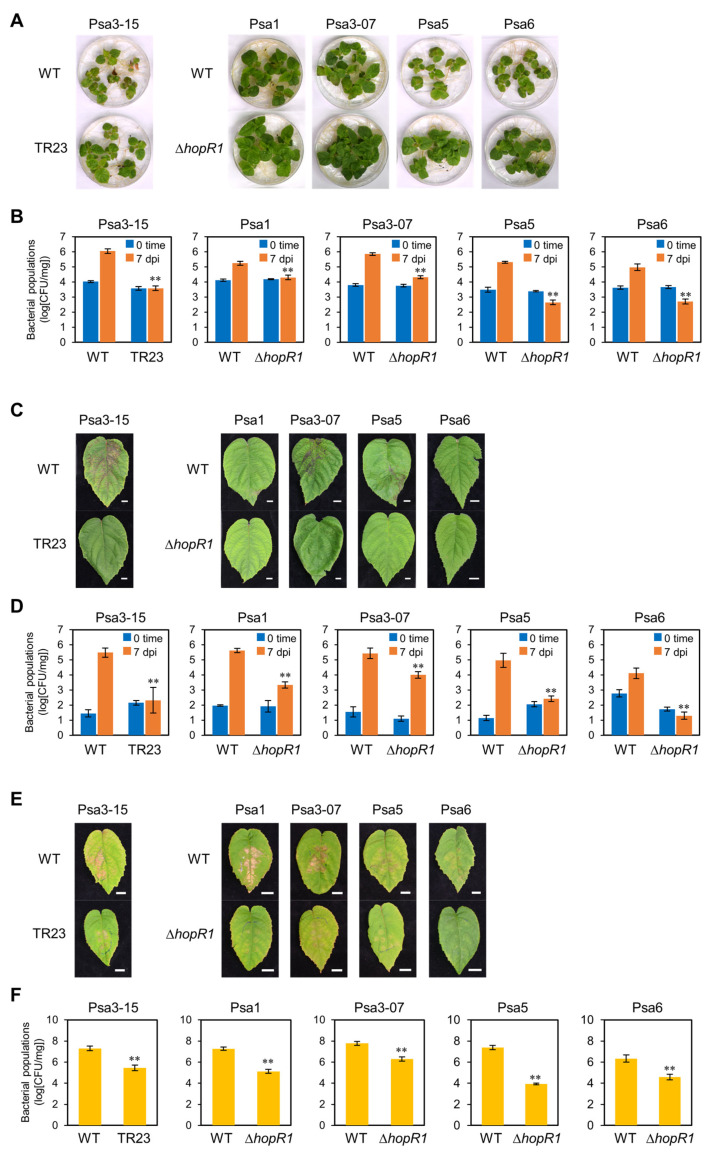
Disease symptoms and bacterial populations in kiwifruit inoculated with *Pseudomonas syringae* pv. *actinidiae* (Psa) WT and Psa biovar Δ*hopR1* mutants. Disease symptoms (**A**) and bacterial populations (**B**) in kiwifruit seedlings flood-inoculated with Psa WT and Psa biovar Δ*hopR1* mutants (1 × 10^8^ CFU/mL) containing 0.025% SilwetL-77. Disease symptoms (**C**) and bacterial populations (**D**) in kiwifruit plants spray-inoculated with Psa WT and Psa biovar Δ*hopR1* mutants (1 × 10^8^ CFU/mL) containing 0.025% SilwetL-77. Disease symptoms (**E**) and bacterial populations (**F**) in kiwifruit plants syringe-inoculated with Psa WT and Psa biovar Δ*hopR1* mutants (5 × 10^6^ CFU/mL). The leaves were photographed at 7 dpi. Vertical bars indicate the standard error for three independent experiments. Asterisks indicate a significant difference between Psa WT and Δ*hopR1* mutant in a *t* test (** *p* < 0.01). Scale bar shows 1 cm.

Psa is currently classified into five biovars, namely biovars 1, 2, 3, 5, and 6 (Table 2). So far, Psa biovars 1, 3, 5, and 6 have been found in Japan, and these biovars have *hopR1* gene [52]. To assess the importance of HopR1 in virulence, we constructed a *hopR1* mutant (Δ*hopR1*) in each biovar. For construction of Δ*hopR1* mutant strains, we used four biovars, Psa1, Psa3-07 (same type as reference strain (ICMP18884; [53]), Psa5, and Psa6 (Table 2). Growth curve assays for each Psa WT and *hopR1* mutant in LB broth showed that bacterial growth did not differ significantly at 24 h (Supplementary Figure S8A–E). Next, to investigate if HopR1 contributes to virulence in these four biovars, we conducted flood-inoculation with kiwifruit seedlings using Psa WT and the *hopR1* mutant series. Consistent with a previous study [44], kiwifruit seedlings inoculated with Psa1, Psa3-07, and Psa3-15 WT exhibited severe disease symptoms such as leaf spots and necrosis; in contrast, seedlings inoculated with Psa5 and Psa6 WT exhibited mild symptoms (Figure 3A). All kiwifruit seedlings inoculated with each *hopR1* mutant biovar exhibited less leaf spot and necrosis at 7 dpi (Figure 3A) compared to each Psa WT. We also investigated whether HopR1 contributes to bacterial multiplication in kiwifruits. All *hopR1* mutant populations were significantly reduced compared to each Psa WT at 7 dpi (Figure 3B). These results indicate that HopR1 universally contributes to Psa virulence on kiwifruit seedlings.

**Table 2:** Bacterial strains and plasmid used in this study.

Bacterial Strain or Plasmid	Relevant Characteristics	Reference or Source
*E. coli* strain		
DH5α	F^−^, λ^−^, φ80d*lacZ*DM15, D(*lacZYA*-*argF*)U169, *deoR*, *recA*1, *endA*1, *hsdR*17 (r_K_^−^, m_K_^+^), *phoA*, *supE*44, *thi-*1, *gyrA*96, *relA*1	Takara Bio, Kusatsu, Japan
S17-1	F^−^, *thi*, *pro*, *hsdR*, *hsdM*^+^, *recA* [chr::*RP4-2-Tc::Km::Tn7*]	[54]
*P. syringae* pv. *actinidiae* (Psa)		
Psa1	Psa biovar 1 wild-type, Nal^r^	MAFF 613022
Psa1-Δ*hopR1*	Psa biovar 1 Δ*hopR1* mutant, Nal^r^	This study
Psa3-07	Psa biovar 3 wild-type, Nal^r^	MAFF 212107
Psa3-07-Δ*hopR1*	Psa biovar 3 Δ*hopR1* mutant, Nal^r^	This study
Psa3-15	Psa biovar 3 wild-type, Nal^r^	MAFF 212115
Psa3-VR series	Whole genome Tn*5* transposon library, Nal^r^, Km^r^, Cm^r^	This study
Psa5	Psa biovar 5 wild-type, Nal^r^	MAFF 212056
Psa5-Δ*hopR1*	Psa biovar 5 Δ*hopR1* mutant, Nal^r^	This study
Psa6	Psa biovar 6 wild-type, Nal^r^	MAFF 212133
Psa6-Δ*hopR1*	Psa biovar 6 Δ*hopR1* mutant, Nal^r^	This study
Plasmid		
pBSLC1	Transposon vector constructed by ligation of pBSL118 and pHSG396 at *Eco*R I site, Amp^r^, Km^r^, Cm^r^	[55]

Notes: Amp^r^ ampicillin resistance, Cm^r^ chloramphenicol resistance, Km^r^ kanamycin resistance, Nal^r^ nalidixic acid resistance.

Then, to investigate if HopR1 contributes to Psa3 virulence in adult kiwifruit plants, we spray-inoculated soil-grown kiwifruit plants with Psa WT and the *hopR1* mutants. Kiwifruit leaves inoculated with Psa3-07 and Psa3-15 WT exhibited severe symptoms such as leaf spots and necrosis on mature leaves, whereas Psa1, Psa5, and Psa6 WT caused mild symptoms on mature leaves (Figure 3C). Conversely, severe symptoms were not observed on *hopR1* mutant inoculated leaves. Consistent with disease development, the *hopR1* mutant populations were significantly reduced compared to Psa WT (Figure 3D). These results also indicate that HopR1 universally contributes to virulence in each Psa biovar on mature kiwifruit plants. 

Finally, we conducted a syringe-infiltration to determine whether HopR1 is important for apoplastic multiplication to cause disease. Kiwifruit leaves infiltrated with Psa WT showed necrosis at 7 dpi. Kiwifruit leaves inoculated with *hopR1* mutants in Psa1, Psa5, and Psa6 showed much less necrosis compared to the corresponding WT (Figure 3E). Interestingly, kiwifruit leaves inoculated with *hopR1* mutants in Psa3-07 and Psa3-015 still showed necrosis, although less severe symptoms compared to the corresponding WT (Figure 3E). The *hopR1* mutant populations were significantly reduced compared to each Psa WT at 7 dpi (Figure 3F). Interestingly, the *hopR1* mutant populations in Psa3-07 and Psa3-015 were greater among the *hopR1* mutants, although Psa3 *hopR1* mutant populations were reduced compared to the corresponding WT (Figure 3F). These results indicate that HopR1 universally contributes to Psa virulence, but the contribution differs depending on the Psa biovar. More specifically, in Psa3, HopR1 might contribute to virulence at the plant surface before multiplication in the apoplast, although in biovars Psa1, Psa5, and Psa6, HopR1 might contribute to virulence both at the plant surface and apoplastic multiplication. Therefore, we hypothesized that *hopR1* shows different expression patterns for each Psa biovar during infection.

### 2.7. HopR1 Regulates Stomatal-Based Defense and Defense-Related Gene Expression in Kiwifruits

**Figure 4:**
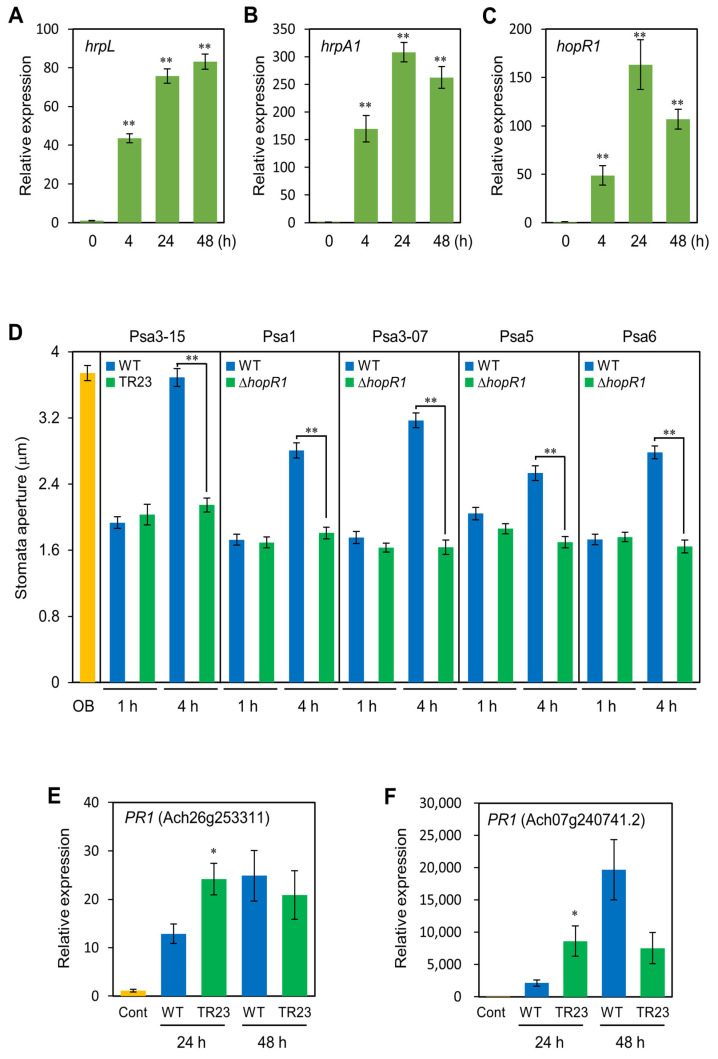
Expression profiles of the *Pseudomonas syringae* pv. *actinidiae* (Psa) genes during kiwifruit seedling infection and kiwifruit defense responses to Psa WT and Psa Δ*hopR1* mutants. Expression profiles of Psa3 *hrpL* (**A**), *hrpA1* (**B**), and *hopR1* (**C**) genes in in flood-inoculated kiwifruit seedlings with the Psa3-15 WT (1 × 10^8^ CFU/mL) at 0, 4, 24, and 48 h. Total RNA was extracted for use in real-time quantitative reverse transcription-polymerase chain reaction (RT-qPCR) with gene-specific primer sets (Table S3). Expression was normalized using fructose-bisphosphate aldolase *fbp*, chromosome participating protein *parA*, and *tetR* family transcriptional regulator. Vertical bars indicate the standard error for three biological replicates. Asterisks indicate a significant difference from 0 time in a *t* test (** *p* < 0.01). (**D**). Stomatal aperture width on kiwifruit leaves 1 h and 4 h after dip-inoculation with 1 × 108 CFU/mL of Psa WT and *hopR1* mutants. OB indicates stomatal opening buffer. Asterisks indicate a significant difference from WT in a *t* test (** *p* < 0.01). Expression profiles of kiwifruit defense marker *PR1* genes (**E**,**F**) in flood-inoculated kiwifruit seedlings with the Psa3-15 WT and *hopR1* mutant (1 × 10^8^ CFU/mL) at 24 and 48 h. Total RNA was extracted for use in real-time quantitative reverse transcription-polymerase chain reaction (RT-qPCR) with gene-specific primer sets (Table S3). Expression was normalized using kiwifruit *actin* (*ACT*) and *ubiquitin* (*UBQ*) genes. Vertical bars indicate the standard error for three biological replicates. Asterisks indicate a significant difference from WT in a *t* test (* *p* < 0.05).

We next determined the *hopR1* expression profiles in Psa3-015 during infection. In this assay, kiwifruit seedlings were flood-inoculated with Psa3-015 WT, and at 0, 4, 24, and 48 hpi total RNAs were purified for real-time RT-qPCR [44]. Expression analysis of *hrpL*, *hrpA1,* and *hopR1* was conducted using RNAs from flood-inoculated seedlings. All genes were greatly expressed at 4 hpi (Figure 4A–C), at which point bacteria are supposed to overcome stomatal-based defense. Moreover, these genes also showed great expression at 24 hpi (Figure 4A–C). Therefore, we hypothesized that HopR1 is involved in overcoming both early stages and later infection processes.

Plants are able to respond to bacterial pathogens by actively closing the stomatal pore, the so called stomatal-based defense [24,28,32]. Since we revealed that HopR1 contributes to virulence at the plant surface before multiplication in the apoplast, we next investigated whether HopR1 facilitates stomatal reopening in kiwifruit by observing the stomatal aperture width of leaves dip-inoculated with Psa WT and *hopR1* mutants at 1 hpi and 4 hpi. Stomatal reopening was observed in kiwifruit inoculated with Psa WT, whereas stomatal reopening was not observed in leaves inoculated with *hopR1* mutants (Figure 4D). These results suggest that HopR1 has an important role in overcoming stomatal-based defense in Psa early virulence in kiwifruit leaves.

To evaluate the effect of HopR1 on defense-related gene expression in kiwifruit leaves, we next investigated *PR1* gene expression profiles in response to Psa3 WT and TR23 (*hopR1* mutant). Kiwifruit *PR1* genes showed significantly greater expression levels at 24 h in response to TR23 compared to Psa3 WT (Figure 4E,F). These results indicate that HopR1 suppresses defense-related gene expression in kiwifruit leaves. 

## 3. Discussion

We developed a rapid, reliable, and high-throughput inoculation method using kiwifruit seedlings to investigate the molecular mechanism of Psa3 infection. We isolated 91 Psa3 mutants showing reduced virulence on kiwifruit seedlings and identified potential virulence factors based on their predicted function (Table 2 and Table S1). We demonstrated that the T3SS/T3SEs have an important role in Psa3 virulence. Our results also indicated that HopR1 contributes to Psa3 virulence on kiwifruit seedlings and mature plants (Figure 2). We also demonstrated that HopR1 contributes to Psa3 virulence on kiwifruit plants in both early and later stages of the infection process by suppressing stomatal-based and apoplastic defenses (Figure 4D–F). The results from *hopR1* mutants in other Psa biovars revealed that HopR1 universally contributes to virulence in all Psa biovars (Figure 3). Our results clearly provide new insights into HopR1 virulence function during Psa infection processes.

We identified 91 Psa3 virulence factors and categorized, based on their predicted function including the T3SS and T3SEs, transporters and cell motility/chemotaxis/adhesion, regulators, primary metabolism such as nucleotide metabolism, amino acid metabolism, carbohydrate metabolism, and lipid metabolism (Table 2 and Table S1). Schreiber et al. [39] identified genes involved in the T3SS, periplasmic glucan biosynthesis, flagellar motility, and amino acid biosynthesis through a high-throughput screening with *P. syringae* pv. *maculicola* ES4326 transposon mutants required for virulence on *Arabidopsis thaliana*. Nearly half of these mutations were in genes associated with the T3SS [39]. Additionally, Brooks et al. [38] conducted a screening of *Pst* DC3000 and *A. thaliana* and found that the mutations disrupted genes involved in the T3SS, the phytotoxin coronatine, and amino acid biosynthesis. Similarly, we also identified genes involved in the T3SS and amino acid biosynthesis (Table 1 and Supplementary Table S1). Brooks et al. [38] identified that around 15% of these genes were related to the T3SS and coronatine biosynthesis, respectively. Similarly, we found around 13% of disrupted genes were related to the T3SS. Sakata et al. [40] conducted a screening for *P. cannabina* pv. *alisalensis* KB2011 Tn*5* mutants by dip-inoculation on cabbage plants. They found transporters and transcriptional regulators as virulence factors in addition to the T3SS and amino acid biosynthesis. Similarly, we found eight mutants in transporters and LysR family transcriptional regulators. Further, Helmann et al. [56] identified 4296 genes in *P. syringae* pv. *syringae* B728a that contributed to the fitness through genome-wide fitness profiling with a randomly barcoded transposon mutant library grown on the leaf surface and in the apoplast of common bean. There were many similarities between their identified genes and ours, such as amino acid metabolism and transport related genes (including serine hydroxymethyltransferase (*glyA-2*), homoserine O-acetyltransferase (*metX*), aminodeoxychorismate/anthranilate synthase component II (*trpE*)), carbohydrate transport and metabolism related genes (including GDP-mannose 4,6-dehydratase (*gmd*)), nucleotide metabolism and transport related genes (including phosphoribosylformylglycinamidine cyclo-ligase (*purM*), and glycosyl transferase). Helmann et al. [56] also measured growth of *Pss* B728a auxotrophic mutant strains. Our results showed nine mutants that did not grow in MMMF medium as partial auxotrophs. There were also similarities between these genes identified in partial auxotroph mutants (Table 1). Since we used dip-inoculation for screening to imitate natural infection, this method led to the identification of virulence factors required during the infection process, regardless of the *Pseudomonas* pathovar or epiphytic and endophytic condition.

Patel et al. [57] identified 58 Psa Tn*5* transposon mutants involved in lipolytic activity and their role in kiwifruit leaf colonization. Consistent with their screening, we also identified virulence genes related to chemotaxis protein, including cell division ATP-binding protein FtsE (*ftsE*), protein disulfide-isomerase (*dsbC*), and dienelactone hydrolase. Chemotaxis is a way for plant-pathogenic bacteria to sense and respond to chemicals released from plant tissues to the leaf surface [58,59,60]. Zhao et al. [61] identified probable pathogenic genes by detecting divergent Psa3 strain mutations using a computational pipeline. There were also several similarities between their identified genes and ours such as filamentous hemagglutinin, FAD-dependent oxidoreductase, ABC transporter, and major facilitator superfamily (MFS) transporter. Jayaraman et al. [62] showed that lipopolysaccharide (LPS) mutants GDP-D-mannose 4,6-dehydratase (Δ*gmd*) in both Psa1 and Psa3 displayed reduced virulence compared to their WT. They characterized a *P. syringae* LPS, common polysaccharide antigen (CPA) locus from Psa1 and Psa3. This locus has genes for L- and D-rhamnose biosynthesis, and an operon coding for ABC transporter subunits, a bifunctional glycosyltransferase, and an O-methyltransferase. We also identified genes such as *gmd*, *sugar ABC transporter*, and *glycosyltransferase*. These results show that not only is the screening system we developed working correctly, but also that this system is able to identify virulence factors efficiently during Psa infection. Although it is necessary to investigate the precise functional analysis of each virulence factor, these similarities and virulence scores suggest their important role in Psa virulence mechanisms.

Although the T3SS/T3SEs had the greatest number of mutant strains showing reduced virulence, the virulence score trends were different (Table 1 and Table S1). Based on virulence score and HR cell death (Supplementary Figure S6), the T3SS/T3SEs mutants were categorized into three groups, T3SS mutants (showed complete pathogenicity impairment with virulence score 0, no HR), T3SS locaters (virulence score 0.67-3 and less HR), and T3SEs (virulence score 0.67–3.3 and WT HR level). Previous studies showed that HrpK1 and HrpH contributed to translocation of T3SEs. They elicited reduced HR cell death and the elicitation activity by the mutants depended on their concentration [48,49]. Consistent with their results, we also demonstrated that T3SS locater mutants elicited a reduced, or the same, level of HR cell death compared to Psa3 WT. The T3SEs, *hopZ3* and *hopAC1* mutants showed, respectively, high virulence scores of 3 and 3.67. In fact, the *hopAC1* gene in Psa3 appears to be disrupted by a transposable element in the *Pseudomonas* genome as well as its gene in *Pst* DC3000 [63]. HopZ3 is involved in survival and growth of *Pss* B728a on leaf surfaces to enzymatically modify host targets [64]. Taken together, HopZ3 and HopAC1 contribute to Psa virulence, partially or during the epiphytic phase at leaf surfaces. 

We identified both *avrE1* and *hopR1* as reduced virulence mutants from our screening (Table 1). AvrE1 and HopR1 were identified as AvrE/DspA/E/HopR superfamily members [35]. Xin et al. [36] demonstrated that the virulence function of HopM1 and AvrE1 can be substituted by supplying water to the apoplast to establish an aqueous living space in plant leaves. Jayaraman et al. [65] showed that HopM1 does not contribute to Psa3 virulence due to a gene truncation, and AvrE1 and HopR1 are required for Psa3 virulence. Further, Jin et al. [66] showed that *Pst* DC3000 AvrE1 targets plant protein phosphatase 2A (PP2A) to disrupt PP2A normal function, which positively regulates early PTI signaling. Taken together, in Psa3, AvrE1 and HopR1 may contribute to establish an aqueous living space in plant leaves. We identified both *avrE1* and *hopR1* mutants from our screening. Kiwifruit seedlings and mature plants inoculated with the *hopR1* mutant showed the least symptoms and multiplication among the T3SEs (Figure 2D). We also demonstrated that the *hopR1* and *avrE1* mutants showed remarkable virulence reduction with spray-inoculation compared to flood-inoculation (Figure 2D). Conversely, there were no significant differences in disease development and bacterial populations between the *hopR1* mutant and other T3E mutants with syringe-inoculation (Figure 2E,F). Further, we demonstrated that HopR1 contributes to Psa virulence on kiwifruit plants in both early and later stages of the infection process by suppressing stomatal-based and apoplastic defenses (Figure 4F). *Pst* DC3000 HopR1 also contributes to PTI suppression in *N. benthamiana* leaves [35]. Although the HopR1 target is still unknown, these effectors probably contribute to suppress PTI as well as to establish an aqueous living space in plant leaves. Taken together, HopR1 might contribute to virulence to suppress PTI at the plant surface or before apoplastic multiplication to cause disease.

In summary, HopR1 contributes to Psa virulence on kiwifruit plants in both early and later stages of the infection process by suppressing stomatal-based and apoplastic defenses.

## 4. Materials and Methods

### 4.1. Plant Materials and Growth Conditions

Kiwifruit (*Actinidiae deliciosa*) cv. ‘Hayward’ plants were used for all experiments. Kiwifruit seeds (100–200) were surface-sterilized in 70% (*v*/*v*) ethanol for 5 min in a Falcon tube (50 mL), then in 5% (*v*/*v*) sodium hypochlorite (FUJI- FILM Wako Pure Chemical Corporation, Osaka, Japan) containing 0.1% (*v*/*v*) Tween 20 (Sigma-Aldrich, St. Louis, MO, USA) for 1 h. Seeds were then washed with sterile distilled H_2_O at least four times and incubated in water at 4 °C overnight. Surface sterilization and washing were repeated, and seeds were germinated on one-half strength MS medium (FUJIFILM Wako Pure Chemical Corporation) containing 1% (*w*/*v*) sucrose and Gamborg vitamins (Sigma-Aldrich) solidified with 0.3% (*w*/*v*) Phytagel (Sigma-Aldrich) in deep Petri plates (100 mm × 25 mm). Four-week-old kiwifruit seedlings were grown at 24 °C with a light intensity of 150–200 μmol/ (m_2_ sec) and 12 h light/12 h dark before use. Eight-week-old kiwifruit plants were grown in soil for spray-inoculation and syringe-inoculation. Tobacco (*Nicotiana tabacum*), cv. Xanthi plants were used for the Psa hypersensitive reaction (HR) cell death assay. Kiwifruit and tobacco plants were grown at 24 ℃ with a light intensity of 200 μEm^−2^s^−1^ and a 16 h light/8 h dark photoperiod.

### 4.2. Bacterial Strains, Plasmids, and Growth Conditions

All bacterial strains and plasmids used in this study are shown in Table 1. *Pseudomonas syringae* pv. *actinidiae* biovar 1 (Psa1; MAFF 613022), biovar 3 (Psa3; MAFF 212115), biovar 3 (Psa3-07; MAFF 212107), biovar 5 (Psa5; MAFF 212056), and biovar 6 (Psa6; MAFF 212133) were a gift from NARO Genebank, Ibaraki, Japan. Psa biovars were used as the pathogenic strains to inoculate kiwifruit plants. All Psa strains were grown at 28 °C on King’s B (KB) [67] agar. For inoculation, Luria–Bertani (LB) [68] broth was used to grow bacterial cultures from plates for 18 h at 28 °C. Before inoculation, bacteria were suspended in sterile distilled H_2_O, and the bacterial cell densities at 600 nm (OD_600_) were measured using a JASCO V-730 spectrophotometer (JASCO, Tokyo, Japan).

### 4.3. Generation of a Psa3 Genomic Tn5 Mutant Library

The transposon was introduced into Psa3 by conjugation with *E. coli* S17-1, which possessed pBSLC1 [52], and the insertion region was integrated into the Psa3 chromosome randomly. Replica plates for all transconjugants were made and used for the inoculation assay.

### 4.4. Plasmid Rescue of Transposon-Integrated Regions and Sequencing Analysis to Identify Insertion Sites

Genomic DNA of the mutants that showed reduced virulence on kiwifruit was purified using a Nucleospin Microbial DNA Kit (Takara Bio, Kusatsu, Shiga, Japan) and digested with *Hin*d III, *Xho* I, *Sph* I, *Kpn* I, *Sal* I, *Xba* I, or *Hin*c II (Takara Bio). The resultant DNA was ligated with T4 DNA ligase (Ligation-convenience kit, Nippon Gene, Tokyo, Japan), then introduced into *E. coli* DH5α competent cells. Plasmid DNA was purified from the transformants, and transposon-insertion sites were identified by sequencing with the M13 forward primer. A *Pseudomonas* Genome DB BLAST search (http://www.pseudomonas.com/blast/setnblast, accessed on 14 February 2020) was utilized to identify the function of the mutated genes.

### 4.5. Generation of ∆hopR1 Mutants

The genetic regions containing *hopR1* and the surrounding regions were amplified using PCR primer sets (for *hopR1*) that were designed based on the registered sequence of Psa biovar3 (ICMP 18884) with PrimeStar HS DNA polymerase (Takara Bio). Then, dA was added to the 3′ end of the PCR product with 10× A-attachment mix (TOYOBO, Osaka, Japan). The resultant DNA was inserted into the pGEM-T Easy vector (Promega, Madison, WI, USA). The recombinant plasmid DNA was then used to obtain pGEM-*hopR1* as templates, and inverse PCR was carried out using a primer set (for *hopR1*) to delete a *hopR1* open reading frame. Then, the PCR product and template DNA were digested with *Bam*H I and *Dpn* I. The resultant DNA was self-ligated with T4 DNA ligase (Ligation-Convenience kit, Nippon Gene, Tokyo, Japan). The *hopR1*-deleted DNA constructs were introduced into the *Eco*R I site of the mobilizable cloning vector pK18*mobsacB* [69]. The resulting plasmids containing the DNA fragment lacking hopR1 were then used to transform *E. coli* S17-1. The deletion mutant was obtained by conjugation and homologous recombination [54]. Transconjugants were selected on KB agar containing 30 μg/mL of kanamycin (Km).

### 4.6. Growth Curve Assay

Psa strains including the wild type, Tn*5* transposon mutants, and ∆*hopR1* mutants were grown at 28 °C for 24 h in LB broth. The strain suspensions were adjusted to an OD_600_ of 0.1 with fresh LB broth, and the bacterial growth dynamics were measured at OD_600_ for 24 h.

### 4.7. Screening Methods

To assay for disease on kiwifruit seedlings, 3000 Psa3 Tn*5* mutants were dip-inoculated on 4-week-old kiwifruit seedlings. Briefly, 10 mL of Psa3 Tn*5* mutant bacterial suspension (OD600 of 0.2) in sterile distilled H_2_O containing 0.025% (*v*/*v*) Silwet L-77 (OSI Specialties Inc., Danbury, CT, USA) was prepared in a 15 mL falcon tube. Two of the 4-week-old kiwifruit seedlings were transferred to the tube for dip-inoculation, then, incubated for 2–3 min at room temperature. Seedlings were transferred to a new one-half strength MS plate, and plates were sealed with 3 M Micropore 2.5 cm surgical tape. Then, disease symptoms were observed up to fourteen-day post-inoculation (dpi). Mutant strains that caused little or no leaf spot were chosen for further analyses. A total of 225 mutants showed reduced virulence on kiwifruit seedlings during the first screening. A second screening was conducted using a flood-inoculation method [44]. Six seedlings were evaluated, and a virulence score assigned by counting infected seedlings, and each experiment was repeated at least three times. Ninety-one mutants showed reduced virulence on kiwifruit seedlings from the second screening and the mutated genes were determined.

### 4.8. Bacterial Inoculation Methods

A flood-inoculation method was used to observe kiwifruit seedling disease symptoms with Psa. Briefly, 40 mL of bacterial suspension at 1 × 10^8^ CFU/mL (OD_600_ of 0.2) in sterile distilled H_2_O containing 0.025% (*v*/*v*) Silwet L-77 (OSI Specialties Inc., Danbury, CT, USA) was dispensed onto a plate containing six 4-week-old kiwifruit seedlings, and the plates were incubated for 2–3 min at room temperature. After the bacterial suspension was removed by decantation, plates containing inoculated seedlings were sealed with 3 M Micropore 2.5 cm surgical tape (3 M, St. Paul, MN, USA) and incubated at 22 °C with a light intensity of 150–200 μmol/ (m^2^sec) and 12 h light/12 h dark cycle. Symptoms were observed at 7- and 14-days post-inoculation (dpi). In each experiment, more than six seedlings were evaluated, and each experiment was repeated at least three times. For spray-inoculation, 6-week-old kiwifruit plants were sprayed to runoff with a bacterial suspension at 1 × 10^8^ CFU/mL (OD_600_ of 0.2) in sterile distilled water containing 0.025% Silwet L-77. The plants were then incubated in growth chambers at approximately 100% RH for the first 24 h, then at approximately 70% RH for the rest of the experiment. The inoculated plants were observed at 7 dpi for symptom development. 

To determine bacterial growth in kiwifruit seedlings or leaves, we measured the internal bacterial population at 7 dpi. Inoculated seedlings or leaves were collected, and the inoculated plants were weighed. Seedlings or leaves were then surface sterilized with 10% H_2_O_2_ for 3 min, then washed three times with sterile distilled water. Plants were then homogenized in sterile distilled water, and the diluted samples were plated onto solid KB agar medium. Bacterial colony forming units (CFU) were normalized as CFU/mg using the total inoculated leaf or seedling mass. The bacterial population at 0 day was estimated using leaves harvested 1 h post-inoculation (hpi). The bacterial populations were evaluated in three independent experiments. For syringe-inoculation, bacteria were suspended at a final concentration of 5 × 10^6^ CFU/mL (OD_600_ of 0.01) and infiltrated with a 1 mL blunt syringe into leaves. The plants were then incubated at 70–80% RH for the rest of the experimental period. Leaves were removed and photographed at 5 days post inoculation. The internal bacterial population was measured after syringe-inoculation. Leaf discs were harvested using a 3.5 mm-diameter cork-borer from syringe-infiltrated leaf zones. Leaf extracts were homogenized in sterile distilled water, and diluted samples were plated onto solid KB agar medium at 7 dpi. After dilution sample plating, the bacterial colony forming units (CFUs) were counted and normalized as CFU per milligram or CFU per cm^2^, using the total leaf weight or leaf square meters. The bacterial populations were evaluated in at least three independent experiments. 

To analyze Psa3-induced HR cell death in tobacco leaves, bacterial suspensions (5 × 10^7^ CFU/mL) of Psa3 WT and virulence mutants were prepared and infiltrated into leaves using a 1 mL needleless syringe. HR cell death was observed 24 h after infiltration.

### 4.9. Real-Time Quantitative RT-PCR

For Psa3 gene expression profiles in culture or during infection, we incubated Psa3 in LB broth for 3 h or flood-inoculated kiwifruit seedlings and incubated for 24, 48, and 72 h. For expression profiles in culture conditions, Psa3 was grown in LB broth for 24 h, then adjusted to an OD_600_ of 0.1 with fresh LB broth and grown for 3 h to investigate the exponential phase expression profiles. Total RNA was extracted from kiwifruit seedlings by an ultra-sonication method as described in [44]. Briefly, 6 seedlings were collected, immediately submerged in 5 mL of RNAlater Stabilization Solution (Thermo Fisher Scientific, Waltham, MA, USA), sonicated for 7 min, then seedlings were removed from the solution. The bacterial cells in the suspension were harvested by centrifugation at 12,000 rpm for 2 min, and cell pellets were used for subsequent purification. Total RNA was extracted using Reliaprep (Promega) according to the manufacturer’s protocol. For gene expression profiles of kiwifruit defense-related genes, four-week-old kiwifruit seedlings were flood-inoculated with Psa3 WT and hopR1 mutant. At 24 and 48 h after inoculation, total RNA was extracted from the inoculated kiwifruit seedlings and purified using Maxwell^®^ RSC Instrument (Promega) according to the manufacture’s protocol. Total RNA (2 µg) was treated with gDNA Remover (TOYOBO) to eliminate genomic DNA, and the DNase-treated RNA was reverse transcribed using the ReverTra Ace qPCR RT Master Mix (TOYOBO). The cDNA (1:20) was then used for qRT-PCR using the primers shown in supplementary Table S1 with THUNDERBIRD^®^ SYBR qPCR Mix (TOYOBO) on a Thermal Cycler Dice Real Time System (Takara Bio). Three genes encoding fructose-bisphosphate aldolase, chromosome participating protein ParA, and a TetR family transcriptional regulator were used for normalizing the Psa results [70]. Kiwifruit *actin* (*ACT*) and *ubiquitin* (*UBQ*) genes were used for normalizing the kiwifruit results [71,72].

### 4.10. Stomatal Assay

Kiwifruit plants were grown in soil for around 6 weeks after germination. Psa WT and *hopR1* mutants were grown at 28 °C for 24 h on KB agar, then suspended in distilled water to an OD_600_ of 0.2 (1 × 10^8^ CFU/mL). Kiwifruits leaves were floated on stomatal opening buffer (10 mM MES-KOH, 20 mM KCl, pH 6.3). Four hours after treatment, kiwifruits leaves were dip inoculated with Psa WT and *hopR1* mutants. Dip-inoculated kiwifruits leaves were directly imaged at 1 hpi and 4 hpi using a Nikon optical microscope (Eclipse 80i). The aperture width of at least 100 stomata was measured. The average and standard error for the stomatal aperture width were calculated. The stomatal apertures were evaluated in at least three independent experiments.

## Figures and Tables

**Table 1:** Classification and characteristics of Psa3 transposon disruption mutants.

Classification	Mutant	Locus	Description	Gene Name	Virulence Score	Growth in MMMF
Amino acid metabolism and transport	TJ35	IYO_RS04020	Serine hydroxymethyltransferase	*glyA-2*	0	-
	TK40	IYO_RS23580	Ketol-acid reductoisomerase		0	-
	TK18	IYO_RS25805	Homoserine O-acetyltransferase	*metX*	1	-
	TQ26	IYO_RS19640	1-aminocyclopropane-1-carboxylate deaminase		1.33	+
	TAk13	IYO_RS29530	Aminodeoxychorismate/anthranilate synthase component II	*trpE*	3	+
Carbohydrate transport and metabolism	TAr22	IYO_RS06400	6-phosphogluconolactonase	*pgl*	0.67	-
	TBt16	IYO_RS10680	Transaldolase	*tal*	1	+
	TAc31	IYO_RS02340	Aminoglycoside phosphotransferase		1.33	-
	TBc20	IYO_RS23010	GDP-mannose 4,6-dehydratase	*gmd*	2	+
	TAt22	IYO_RS10265	Aconitate hydratase	*acnA*	2.67	-
Cell motility/Chemotaxis/Adhesion	TA15	IYO_RS28990	Filamentous hemagglutinin		1	+
	TS01	IYO_RS09885	Fagellar basal body rod protein FlgF	*flgF*	1.33	+
	TAh35	IYO_RS13580	Chemotaxis protein		2	+
	TAg29	IYO_RS23025	Methyl-accepting chemotaxis protein		2.67	+
	TAw30	IYO_RS09875	Flagellar hook protein FlgE	*flgE*	2.67	+
	TAv34	IYO_RS26210	Fimbrial protein	*pilQ*	3.33	+
	TV06	IYO_RS18685	Chemotaxis protein		3.67	+
DNA processing and modification	TN40	IYO_RS11935	Group II intron maturase		1.67	+
	TBw17	IYO_RS29390	Transposase		2	+
	TAc15	IYO_RS29385	Integrase		2.33	+
	TAz17	IYO_RS21055	DEAD/DEAH box helicase		2.33	+
	TAl20	IYO_RS00095	Serine recombinase		2.67	+
	TBj10	IYO_RS05170	Integrase		2.67	+
	TZ39	IYO_RS00125	Type III restriction protein res subunit		2.67	+
	TAj16	IYO_RS27700	Transposase		3	+
	TW16	IYO_RS16675	Integrase		3	+
Glycosyl transferase	TAc09	IYO_RS22975	Glycosyl transferase		2.33	+
	TAm12	IYO_RS22990	Glycosyl transferase family 1		2.33	+
Hypothetical protein	TBf09	IYO_RS29775	Hypothetical protein		1.67	+
	TY14	IYO_RS19470	Hypothetical protein		1.67	+
	TBa33	IYO_RS20550	Hypothetical protein		2	+
	TU31	IYO_RS18120	Hypothetical protein		2.33	+
	TU37	IYO_RS23615	Hypothetical protein		2.33	+
	TAt06	IYO_RS07375	Hypothetical protein		2.67	+
	TAk12	IYO_RS14405	Hypothetical protein		3	+
	TM22	IYO_RS26985	Hypothetical protein		3.33	+
Lipid metabolism and transport	TBj05	IYO_RS15545	Peptidyl-prolyl cis-trans isomerase		3	+
	TAj03	IYO_RS22005	1-acyl-sn-glycerol-3-phosphate acyltransferase		3.33	+
	TBf32	IYO_RS00500	Acetyltransferase	*dsbB*	3.33	+
	TBo40	IYO_RS07835	Acetyltransferase		3.33	+
Membrane	TC07	IYO_RS00540	Membrane protein		2.33	+
	TBp22	IYO_RS20435	Membrane protein		3	+
	TC14	IYO_RS07875	Membrane protein		2	+
Nucleotide metabolism and transport	TBk15	IYO_RS08710	Phosphoribosylformylglycinamidine cyclo-ligase	*purM*	0.67	+
	TV14	IYO_RS24905	23S rRNA pseudouridine synthase D	*rluD*	1	+
	TX17	IYO_RS20385	FAD-dependent 5-carboxymethylaminomethyl-2-thiouridine(34) oxidoreductase MnmC	*mnmC*	1.67	+
	TAd31	IYO_RS05655	Pyrimidine utilization protein D		2	+
	TBb34	IYO_RS25270	Ribonuclease R	*vacB*	2	+
	TAm23	IYO_RS27525	Cell division ATP-binding protein FtsE	*ftsE*	3	+
	TBh17	IYO_RS19355	Endonuclease/exonuclease/phosphatase		3	+
	TBl30	IYO_RS08955	Cytidylate kinase	*cmk*	3	+
	TAr33	IYO_RS18310	23S rRNA (guanine(2445)-N(2))/(guanine(2069)-N(7))- methyltransferase		3.33	+
	TBv24	IYO_RS21455	tRNA 5-methoxyuridine(34)/uridine 5-oxyacetic acid(34) synthase CmoB		3.33	+
Others	TAz15	IYO_RS16115	Monooxygenase		2	+
	TBc29	IYO_RS29540	Coenzyme F390 synthetase		2	+
	TY16	IYO_RS15475	N-acetyltransferase		2	+
	TH14	IYO_RS15735	Alcohol dehydrogenase		2.67	+
Peptidase	TT37	IYO_RS02615	Peptidase M23		1.33	+
	TAc18	IYO_RS27100	Peptidase M20		2	+
	TBt26	IYO_RS12305	Zn-dependent hydrolase		3.33	+
Signal transduction/ Transcriptional regulator	TR05	IYO_RS11995	LysR family transcriptional regulator	*lysR*	0.67	+
	TX21	IYO_RS04255	Sensor domain-containing phosphodiesterase		2	+
	TAa14	IYO_RS06430	DNA-binding response regulator		2.67	+
	TAa40	IYO_RS16240	Two-component sensor histidine kinase	*colS*	3.33	+
	TZ32	IYO_RS07365	Hybrid sensor histidine kinase/response regulator		3.33	+
	TA08	IYO_RS04485	DNA-binding transcriptional activator OsmE	*osmE*	4	+
	TE04	IYO_RS21745	Sigma-54 dependent transcriptional regulator/ response regulator		4	+
Sulfur metabolism	TC13	IYO_RS00620	Pyridine nucleotide-disulfide oxidoreductase		3.67	+
Type III secretion system/Type III secretion effector	TAh32	IYO_RS06840	Type III secretion system export apparatus switch protein	*hrcU*	0	+
	TAl39	IYO_RS06775	Sigma-54-dependent Fis family transcriptional regulator	*hrpR*	0	+
	TBi11	IYO_RS06800	Type III secretion inner membrane ring protein	*hrcJ*	0	+
	TD35	IYO_RS06775	Sigma-54-dependent Fis family transcriptional regulator	*hrpR*	0	+
	TJ40	IYO_RS06855	Type III secretion system export apparatus protein	*hrcR*	0	+
	TAw18	IYO_RS06905	Type III helper protein HrpK1	*hrpK1*	0.67	+
	TR23	IYO_RS24135	Type III effector HopR1	*hopR1*	0.67	+
	TBw35	IYO_RS06765	Type III effector AvrE1	*avrE1*	2.33	+
	TAd35	IYO_RS06770	Lytic transglycosylase	*hrpH*	2.67	+
	TBr13	IYO_RS25475	Type III effector HopAC1	*hopAC1*	3	+
	TM33	IYO_RS28930	Avirulence protein HopZ3	*hopZ3*	3.67	+
Transporters	TAy25	IYO_RS23000	Sugar ABC transporter ATP-binding protein		1	-
	TP3	IYO_RS16960	Ectoine/hydroxyectoine ABC transporter permease subunit EhuC		1.33	+
	TBo16	IYO_RS15485	OmpA family protein		1.67	+
	TW19	IYO_RS02405	Autotransporting lipase, GDSL family		1.67	-
	TL23	IYO_RS11430	AI-2E family transporter		2.33	+
	TY11	IYO_RS21760	MFS transporter		2.67	+
	TBr15	IYO_RS01165	ABC transporter ATP-binding protein		3	+
	TBu09	IYO_RS27805	Sulfonate ABC transporter	*ssuC*	3.33	+
Type IV secretion system	TBr28	IYO_RS07305	Protein disulfide-isomerase	*dsbC*	0.33	-
	TAd01	IYO_RS08235	Conjugative coupling factor TraD, PFGI-1 class		2.67	+
Xenobiotics biodegradation and metabolism	TU11	IYO_RS15180	Dienelactone hydrolase		1.33	+
	TBd36	IYO_RS16315	(2Fe-2S)-binding protein	*vanA*	3	+

The Tn*5* insertion sites were determined using a *Pseudomonas* Genome DB BLAST search based on the *Pseudomonas syringae* pv. *actinidiae* ICMP 18884 genome database. Functional category annotations for Psa3 genes are primarily based on COG [48] and KEGG [49] annotations. The Tn*5* mutants were tested for their ability to grow on MMMF minimal medium.

## Data Availability

The data presented in this study are openly available in Supplementary Material here.

## Supplementary Materials

The following supporting information can be downloaded at: https://www.mdpi.com/article/10.3390/plants12010141/s1. Figure S1: Large-scale Psa3 transposon mutagenesis screening using kiwifruit seedlings. (**A**) Planting the seeds on one-half strength MS plates. (**B**) Kiwifruit seedlings two weeks after germination. (**C**) Transferring the seedlings to new one-half strength MS plates with forceps. Seedling incubation at 24 °C with a light intensity of 150–200 μE m^−2^ sec^−1^ and a 12 h light/12 h dark photoperiod. (**D**) Four-week-old kiwifruit seedlings for screening. (**E**) Preparation of Psa3 transposon mutant inoculum (OD_600_ = 0.2). (**F**) Dip-inoculation for two kiwifruit seedlings. (**G**) Transferring the seedlings to new one-half strength MS plates. (**H**) Disease phenotypes of kiwifruit seedlings 14 days after inoculation with Psa3 transposon mutants. Figure S2: Second screening results in kiwifruit seedlings flood-inoculated with Tn*5* transposon mutants (line number from 1 to 1040). Disease symptoms in kiwifruit seedlings flood-inoculated with 1 × 10^8^ CFU/mL (OD_600_ = 0.2) of Psa3 WT and mutants containing 0.025% SilwetL-77. Figure S3: Second screening results in kiwifruit seedlings flood-inoculated with Tn*5* transposon mutants (line number from 1041 to 2080). Disease symptoms in kiwifruit seedlings flood-inoculated with 1 × 10^8^ CFU/mL (OD_600_ = 0.2) of Psa3 WT and mutants containing 0.025% SilwetL-77. Figure S4: Second screening results in kiwifruit seedlings flood-inoculated with Tn*5* transposon mutants (line number from 2081 to 3000). Disease symptoms in kiwifruit seedlings flood-inoculated with 1 × 10^8^ CFU/mL (OD_600_ = 0.2) of Psa3 WT and mutants containing 0.025% SilwetL-77. Figure S5: Bacterial populations in kiwifruit seedlings flood-inoculated with Psa3 WT and mutants selected from the second screening. Bacterial populations in kiwifruit seedlings flood-inoculated with 1 × 10^8^ CFU/mL (OD_600_ = 0.2) of Psa3 WT and mutants from (**A**) line number from 1 to 1040, (**B**) line number from 1041 to 2080 and (**C**) line number from 2081 to 3000 containing 0.025% SilwetL-77. The bacterial populations were obtained by homogenizing the inoculated leaves after surface-sterilization and plating dilutions to selective media at 7 days post-inoculation (dpi). Vertical bars indicate the standard error for three independent experiments. Asterisks indicate a significant difference from the WT and each mutant in a *t* test (* *p*< 0.05, ** *p*< 0.01). Figure S6: HR cell death assay with the type III secretion mutants in tobacco leaves. The leaf areas were infiltrated with type III secretion mutants indicated at 5 × 10^7^ CFU/mL and photographed 1 day post-inoculation (dpi). ++, extensive necrosis; +, reduced necrosis; -, no symptoms. Figure S7: Growth of type III secretion mutants in LB medium. Absorbance at 600 nm was measured in three replicates per strain for 6, 12, and 24 h. Vertical lines indicate the standard deviation. Figure S8: Growth of *hopR1* mutants in each Psa biovar in LB medium. Absorbance at 600 nm was measured in three replicates per strain for 6, 12, and 24 h. (**A**) Psa3-15 WT and TR23. (**B**) Psa1 WT and Δ*hopR1* mutant (**C**) Psa3-07 WT and Δ*hopR1* mutant. (**D**) Psa5 WT and Δ*hopR1* mutant. (**E**) Psa6 WT (blue) and Δ*hopR1* mutant. Table S1: Number of genes within each functional category from the Psa3 transposon disruption screening. Functional category annotations for the Psa3 genes are primarily based on COG [48] and KEGG [49] annotations, with manual additions. Table S2: Defining gene and gene function of Psa3 type III secretion system and effector mutants. Table S3: Primers (5′ to 3′) used in this study. Spreadsheets: The data presented in this study.
