# Supplementary material for: Large–Scale Transposon Mutagenesis Reveals Type III Secretion Effector HopR1 Is a Major Virulence Factor in Pseudomonas syringae pv. actinidiae
Source: Plants (Basel). 2022 Dec 27;12(1):141. doi: 10.3390/plants12010141 (PMC9823363; doi:10.3390/plants12010141)
Supplement: Supplementary file 1 [file plants-12-00141-s001.zip › Supplementary Figures.pdf]

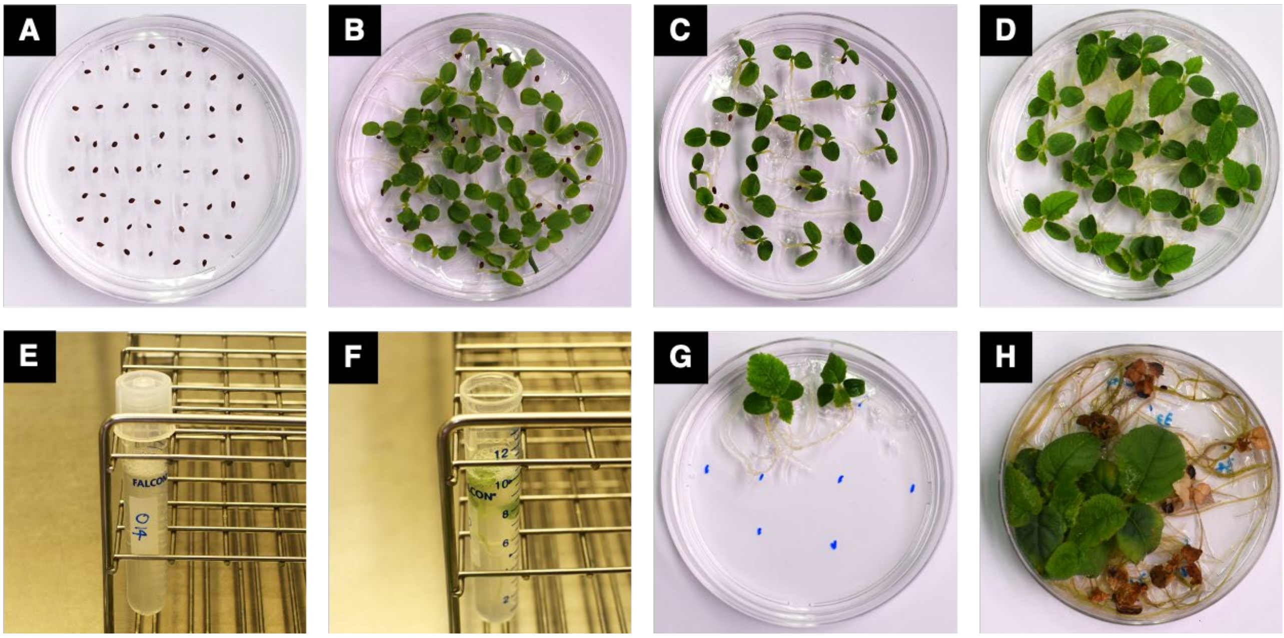

Figure S1: Large-scale Psa3 transposon mutagenesis screening using kiwifruit seedlings. (A) Planting the seeds on one-half strength MS plates. (B) Kiwifruit seedlings two weeks after germination. (C) Transferring the seedlings to new one-half strength MS plates with forceps. Seedling incubation at 24° C with a light intensity of 150-200 μE m^{-2} sec^{-1} and a 12 h light/12 h dark photoperiod. (D) Four-week-old kiwifruit seedlings for screening. (E) Preparation of Psa3 transposon mutant inoculum (OD_{600}=0.2). (F) Dip-inoculation for two kiwifruit seedlings. (G) Transferring the seedlings to new one-half strength MS plates. (H) Disease phenotypes of kiwifruit seedlings 14 days after inoculation with Psa3 transposon mutants.

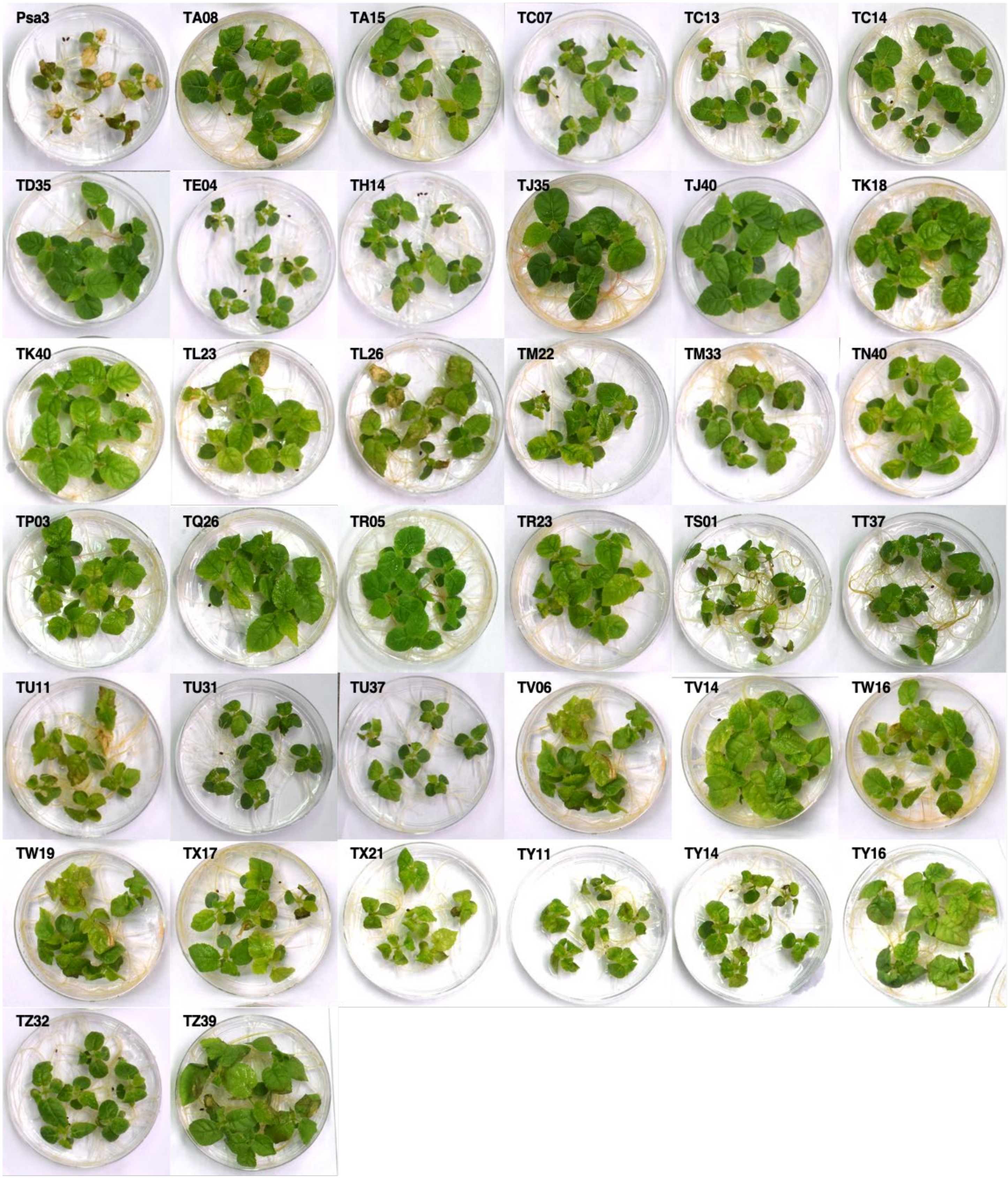

Figure S2: Second screening results in kiwifruit seedlings flood-inoculated with Tn*5* transposon mutants (line number from 1 to 1,040). Disease symptoms in kiwifruit seedlings flood-inoculated with 1 x 10^8 CFU/ml (OD_{600}=0.2) of Psa3 WT and mutants containing 0.025% SilwetL-77.

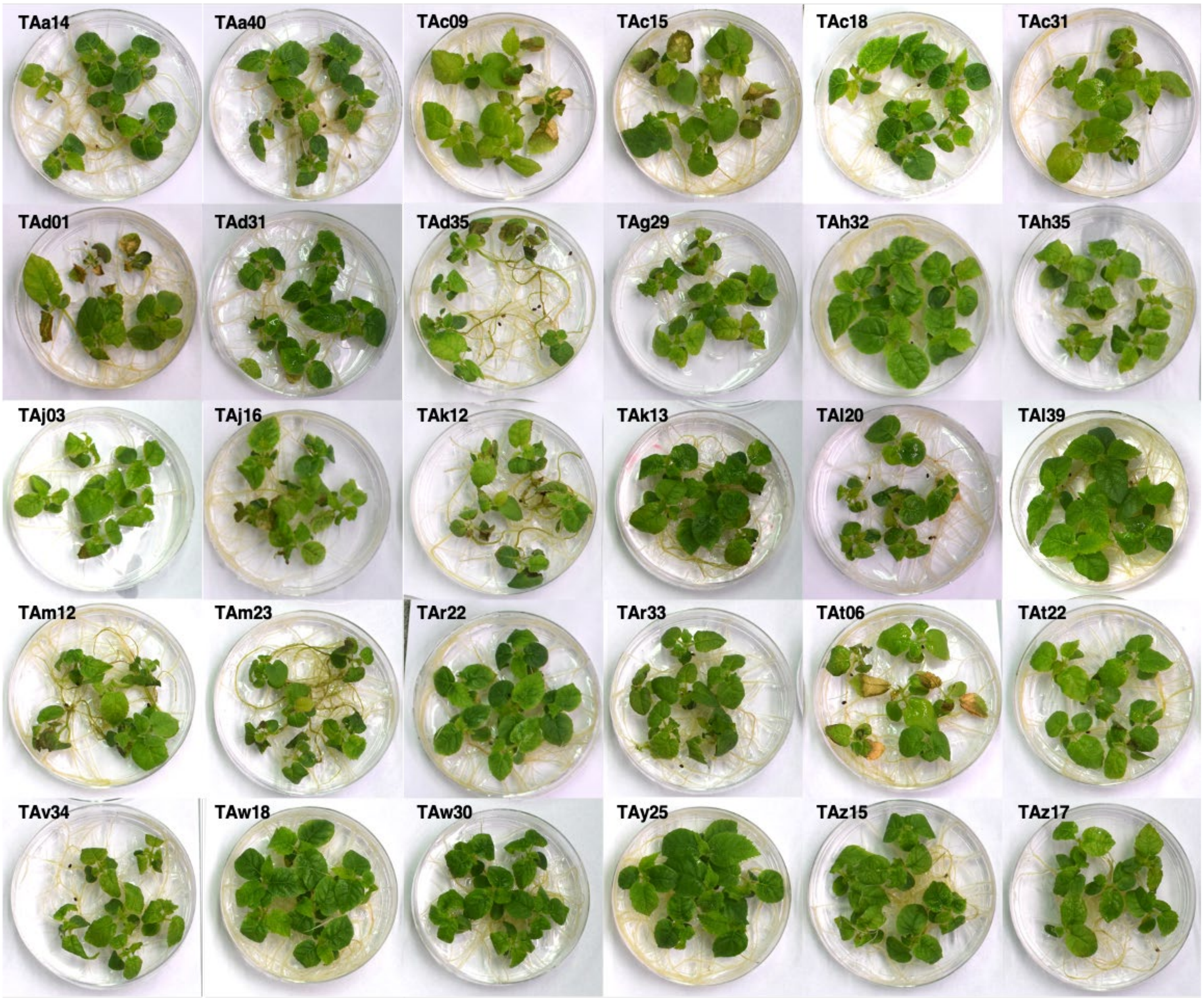

Figure S3: Second screening results in kiwifruit seedlings flood-inoculated with Tn*5* transposon mutants (line number from 1,041 to 2,080). Disease symptoms in kiwifruit seedlings flood-inoculated with 1 x 10^8 CFU/ml (OD_{600}=0.2) of Psa3 WT and mutants containing 0.025% SilwetL-77.

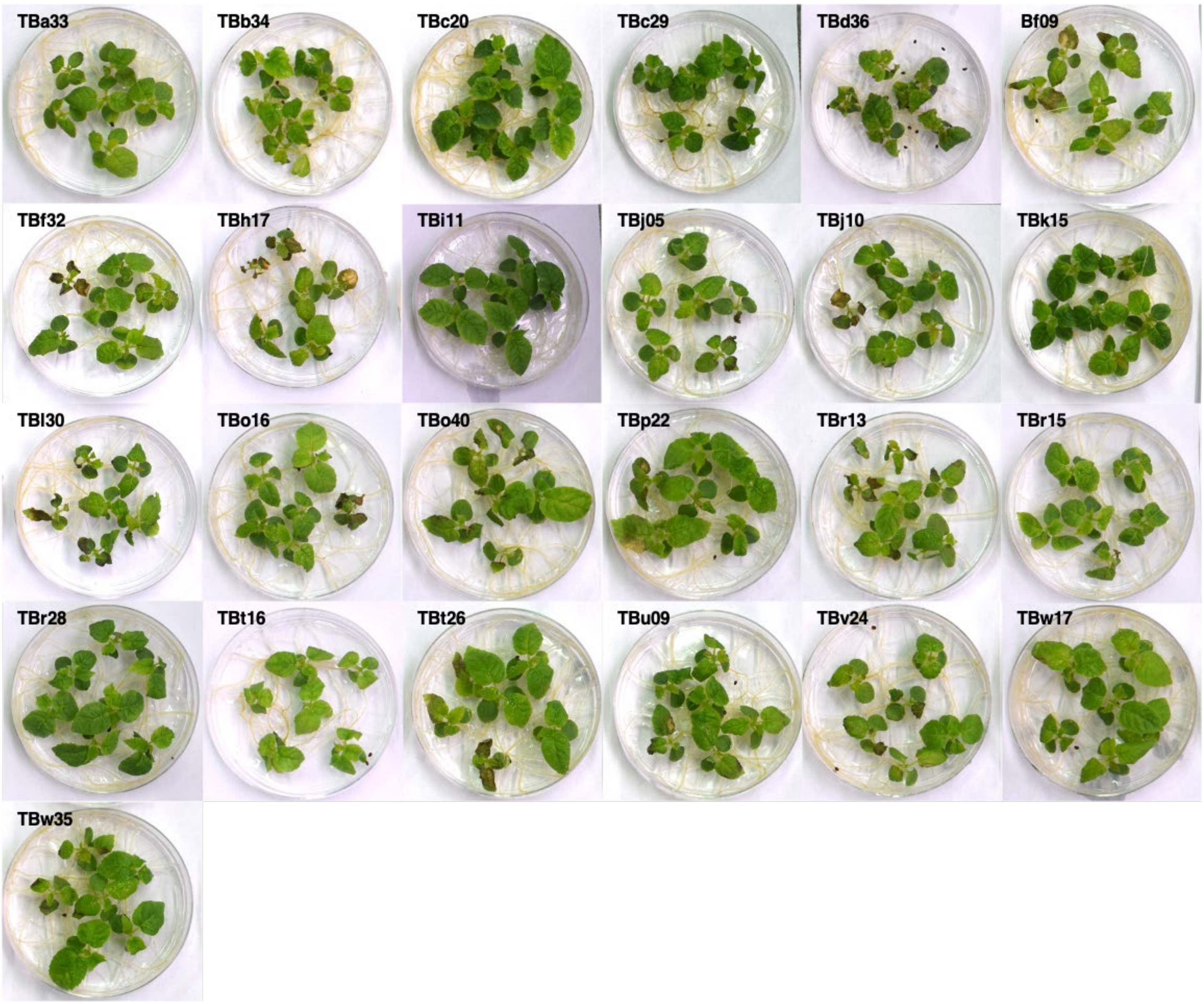

Figure S4: Second screening results in kiwifruit seedlings flood-inoculated with Tn*5* transposon mutants (line number from 2,081 to 3,000). Disease symptoms in kiwifruit seedlings flood-inoculated with 1 x 10^8 CFU/ml (OD_{600}=0.2) of Psa3 WT and mutants containing 0.025% SilwetL-77.

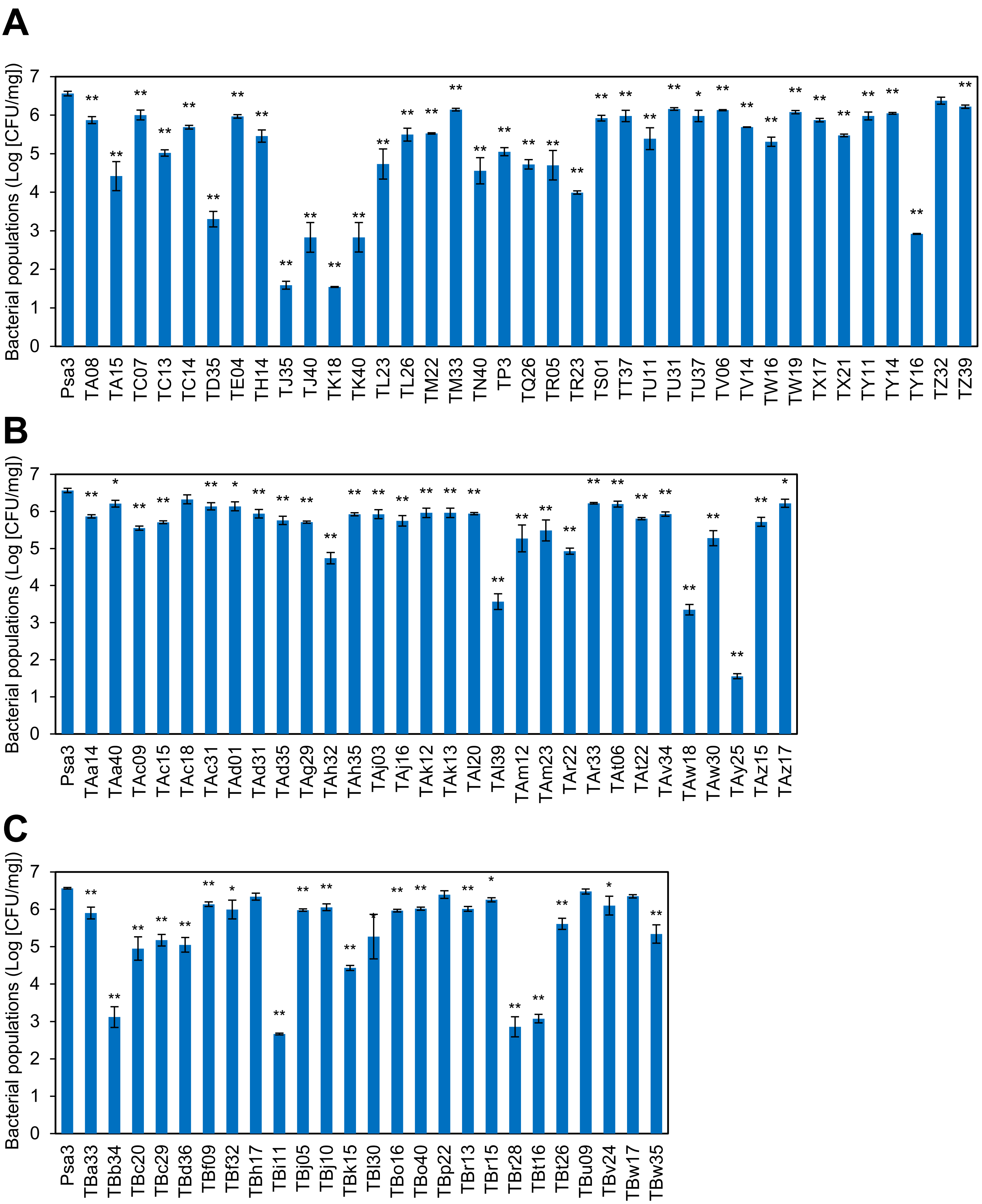

Figure S5: Bacterial population dynamics in kiwifruit seedlings flood-inoculated with Psa3 WT and mutants selected from the second screening. Bacterial populations in kiwifruit seedlings flood-inoculated with 1 x 10^8 CFU/ml (OD_{600}=0.2) of Psa3 WT and mutants from (A) line number from 1 to 1,040, (B) line number from 1,041 to 2,080 and (C) line number from 2,081 to 3,000 containing 0.025% SilwetL-77. The bacterial populations were obtained by homogenizing the inoculated leaves after surface-sterilization and plating dilutions to selective media at 7 days post-inoculation (dpi). Vertical bars indicate the standard error for three independent experiments. Asterisks indicate a significant difference from the WT and each mutant in a *t* test (*p< 0.05, **p< 0.01).

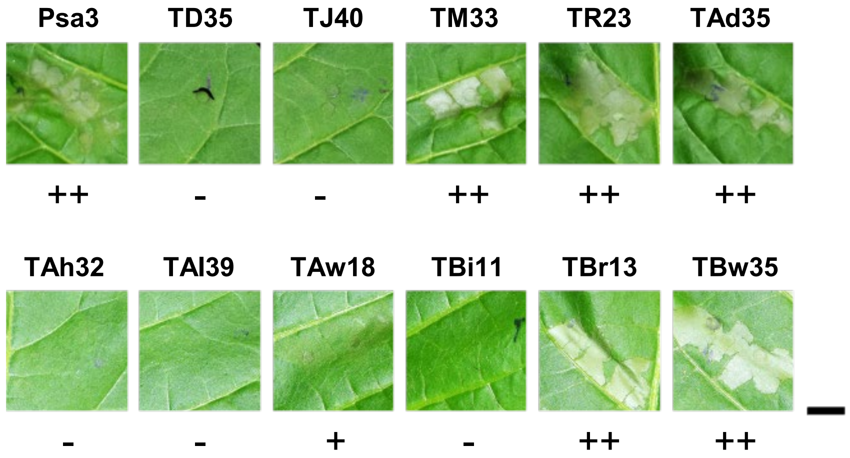

Figure S6: HR cell death assay with the type III secretion mutants in tobacco leaves. The leaf areas were infiltrated with type III secretion mutants indicated at 5 x 10^7 CFU/ml, and photographed 1 day post-inoculation (dpi). ++, extensive necrosis; +, reduced necrosis; -, no symptoms.

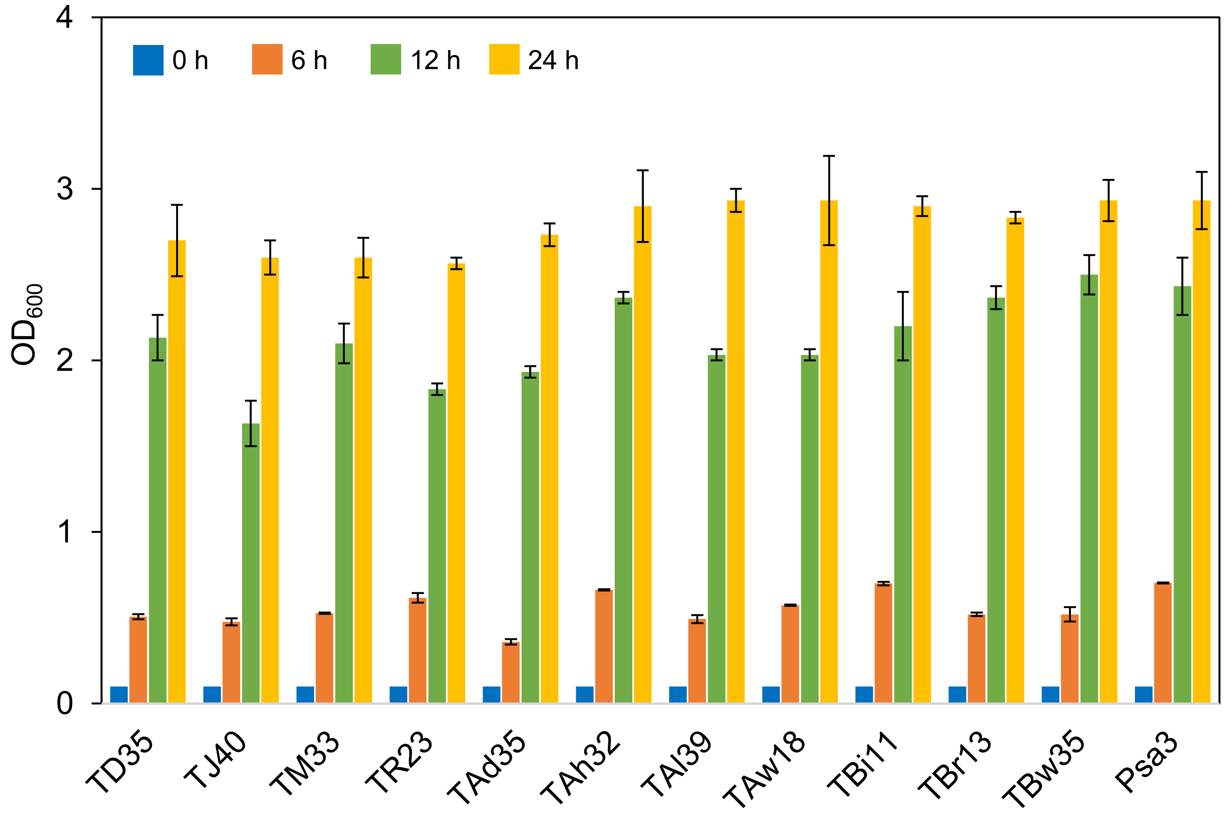

Figure S7: Growth of type III secretion mutants in LB medium. Absorbance at 600 nm was measured in 3 replicates per strain for 6, 12, and 24 hours. Vertical lines indicate the standard deviation.

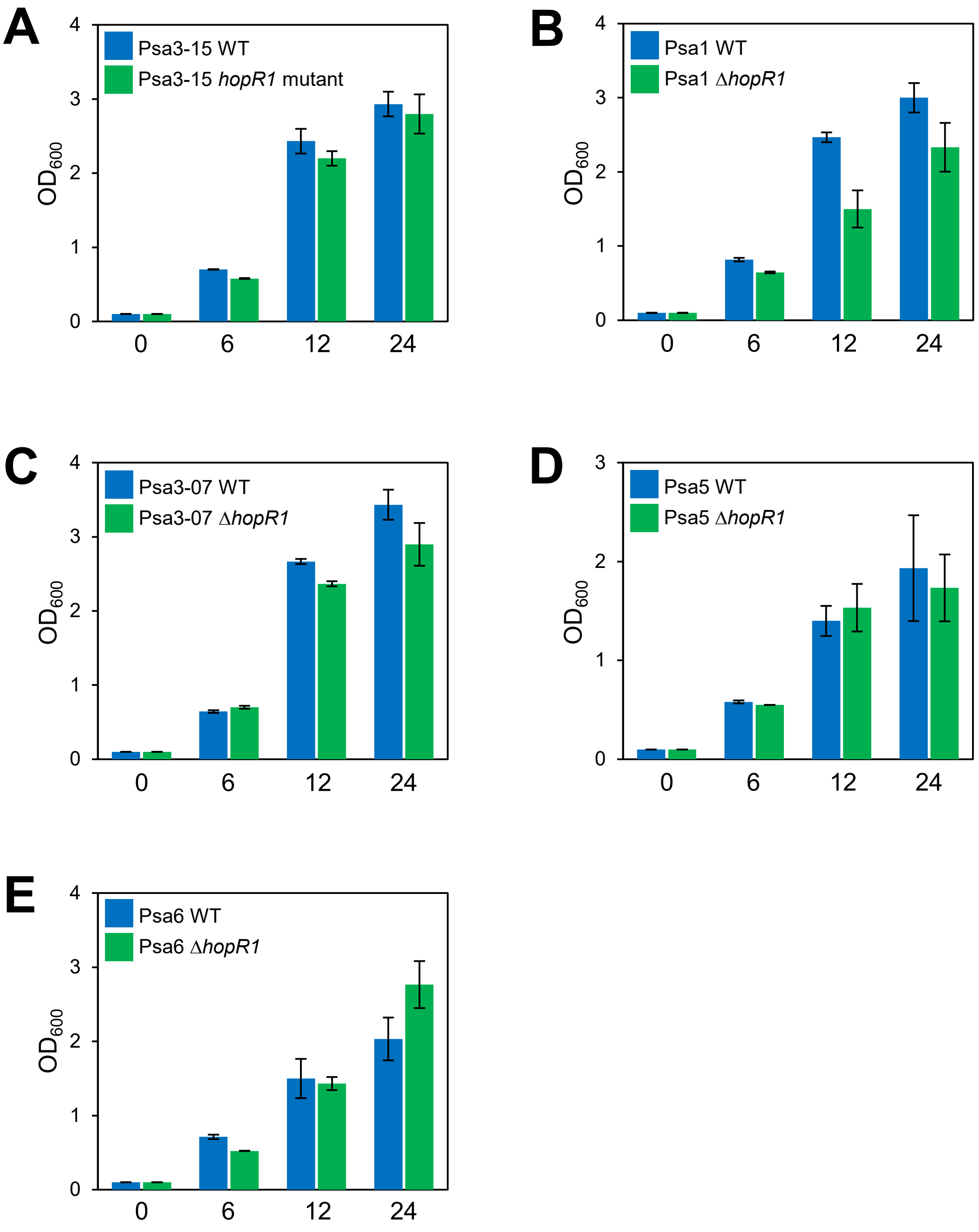

Figure S8: Growth of *hopR1* mutants in each Psa biovar in LB medium. Absorbance at 600 nm was measured in 3 replicates per strain for 6, 12, and 24 hours. (A) Psa3-15 WT and TR23. (B) Psa1 WT and Δ*hopR1* mutant (C) Psa3-07 WT and Δ*hopR1* mutant. (D) Psa5 WT and Δ*hopR1* mutant. (E) Psa6 WT (blue) and Δ*hopR1* mutant.
