# Supplementary material for: Large–Scale Transposon Mutagenesis Reveals Type III Secretion Effector HopR1 Is a Major Virulence Factor in Pseudomonas syringae pv. actinidiae
Source: Plants (Basel). 2022 Dec 27;12(1):141. doi: 10.3390/plants12010141 (PMC9823363; doi:10.3390/plants12010141)
Supplement: Supplementary file 1 [file plants-12-00141-s001.zip › Supplementary Tables.pdf]

Table S1. Number of genes within each functional category (N = 91) from Psa3 transposon disruption screening.

Classification	Number
T3SS /T3SEs	11
Nucleotide metabolism and transport	10
DNA processing and modification	9
Hypothetical protein	8
Transporters	8
Cell motility/Chemotaxis/Adhesion	7
Signal transduction/Transcriptional regulator	7
Amino acid metabolism and transport	5
Carbohydrate transport and metabolism	5
Others	4
Lipid metabolism and transport	4
Membrane	3
Peptidase	3
Glycosyl transferase	2
Type IV secretion system	2
Xenobiotics biodegradation and metabolism	2
Sulfur metabolism	1

Functional category annotations for Psa3 genes are primarily based on COG [72] and KEGG [73] annotations, with manual additions.

Table S2. Characteristics of Psa3 type III secretion system and effector mutants.

Classification	Mutant	Gene locus	Gene	Function	Virulence Score
T3SS	TD35	IYO_RS06775	*hrpR*	Regulator	0
T3SS	TJ40	IYO_RS06855	*hrcR*	Secretion	0
T3SS	TAd35	IYO_RS06770	*hrpH*	Translocator	2.67
T3SS	TAh32	IYO_RS06840	*hrcU*	Secretion	0
T3SS	TAl39	IYO_RS06775	*hrpR*	Regulator	0
T3SS	TAw18	IYO_RS06905	*hrpK1*	Translocator	0.67
T3SE	TBi11	IYO_RS06800	*hrcJ*	Secretion	0
T3SE	TM33	IYO_RS28930	*hopZ3*	Effector	3.67
T3SE	TR23	IYO_RS24135	*hopR1*	Effector	0.67
T3SE	TBr13	IYO_RS25475	*hopAC1*	Effector	3

Table S3. Primer sets used in this study

Gene	Description	Forward primer	Reverse primer	Reference
hopR1	Psa3 Type III effector protein HopR1	TGAGCCAGAGAGGGATGAA	TGATGCGGTGTGCTGATAC	[44]
fbp	Psa3 Fructose-bisphosphate aldolase	TTCTTGCGCCACCTGATTCT	GGATCGAACGCTGGCAGATA	[68]
parA	Psa3 Chromosome partitioning protein ParA	CAACCTGCACGAACTGATGC	GTCCTGCTCATGAGTCGCTT	[68]
tetR	Psa3 TetR family transcriptional regulator	TGACCCATGGCGGTTTCTAC	GCGTGACTCAATGCCTGTTC	[68]
PR1 (Ach26g253311)	Kiwifruit defense marker	ATAGGGACGTTGTCCAATGACG	CAAGTGGTTTGGAGCAGATCG	[69]
PR1 (Ach07g240741.2)	Kiwifruit defense marker	ATCGTAATTGCAAGTGACAAACCAC	GGGAGAATGCTTGCACTATACACAA	This study
ACT	Kiwifruit actin	GTCTGCGATACCAGGGAACAT	GCAGGAATCCATGAGACTACC	[69]
UBQ	Kiwifruit ubiquitin Z	TGCAGATCTTCGTGAAAACC	CCACCACGGAGACGGAGCAC	[70]
